# Supplementary material for: OpenMM 7: Rapid development of high performance algorithms for molecular dynamics
Source: PLoS Comput Biol. 2017 Jul 26;13(7):e1005659. doi: 10.1371/journal.pcbi.1005659 (PMC5549999; doi:10.1371/journal.pcbi.1005659)
Supplement: S1 Source Code — (ZIP) [file pcbi.1005659.s002.zip › OpenMM-7.0.1-Source/docs/api-c++/generated/OpenMM.CustomIntegrator.html]

CustomIntegrator — OpenMM @OPENMM\_MAJOR\_VERSION@.@OPENMM\_MINOR\_VERSION@ documentation


# CustomIntegrator¶

*class* `OpenMM::``CustomIntegrator`¶
:   This is an `Integrator` that can be used to implemented arbitrary, user defined integration algorithms. It is flexible enough to support a wide range of methods including both deterministic and stochastic integrators, Metropolized integrators, and integrators that must integrate additional quantities along with the particle positions and momenta.

    To create an integration algorithm, you first define a set of variables the integrator will compute. Variables come in two types: `CustomIntegrator`. All variables are persistent between integration steps; once a value is set, it keeps that value until it is changed by the user or recomputed in a later integration step.

    Next, you define the algorithm as a series of computations. To execute a time step, the integrator performs the list of computations in order. Each computation updates the value of one global or per-DOF value. There are several types of computations that can be done:

    > - Global: You provide a mathematical expression involving only global variables. It is evaluated and stored into a global variable.
    > - Per-DOF: You provide a mathematical expression involving both global and per-DOF variables. It is evaluated once for every degree of freedom, and the values are stored into a per-DOF variable.
    > - Sum: You provide a mathematical expression involving both global and per-DOF variables. It is evaluated once for every degree of freedom. All of those values are then added together, and the sum is stored into a global variable.
    > - Constrain Positions: The particle positions are updated so that all distance constraints are satisfied.
    > - Constrain Velocities: The particle velocities are updated so the net velocity along any constrained distance is 0.

    Like all integrators, `CustomIntegrator` ignores any particle whose mass is 0. It is skipped when doing per-DOF computations, and is not included when computing sums over degrees of freedom.

    In addition to the variables you define by calling `addGlobalVariable()` and `addPerDofVariable()`, the integrator provides the following pre-defined variables:

    > - dt: (global) This is the step size being used by the integrator.
    > - energy: (global, read-only) This is the current potential energy of the system.
    > - energy0, energy1, energy2, ...: (global, read-only) This is similar to energy, but includes only the contribution from forces in one force group. A single computation step may only depend on a single energy variable (energy, energy0, energy1, etc.).
    > - x: (per-DOF) This is the current value of the degree of freedom (the x, y, or z coordinate of a particle).
    > - v: (per-DOF) This is the current velocity associated with the degree of freedom (the x, y, or z component of a particle’s velocity).
    > - f: (per-DOF, read-only) This is the current force acting on the degree of freedom (the x, y, or z component of the force on a particle).
    > - f0, f1, f2, ...: (per-DOF, read-only) This is similar to f, but includes only the contribution from forces in one force group. A single computation step may only depend on a single force variable (f, f0, f1, etc.).
    > - m: (per-DOF, read-only) This is the mass of the particle the degree of freedom is associated with.
    > - uniform: (either global or per-DOF, read-only) This is a uniformly distributed random number between 0 and 1. Every time an expression is evaluated, a different value will be used. When used in a per-DOF expression, a different value will be used for every degree of freedom. Note, however, that if this variable appears multiple times in a single expression, the
    > - gaussian: (either global or per-DOF, read-only) This is a Gaussian distributed random number with mean 0 and variance 1. Every time an expression is evaluated, a different value will be used. When used in a per-DOF expression, a different value will be used for every degree of freedom. Note, however, that if this variable appears multiple times in a single expression, the
    > - A global variable is created for every adjustable parameter defined in the integrator’s `Context`.

    The following example uses a `CustomIntegrator` to implement a velocity Verlet integrator:

    ```
    CustomIntegrator integrator(0.001);
    integrator.addComputePerDof("v", "v+0.5*dt*f/m");
    integrator.addComputePerDof("x", "x+dt*v");
    integrator.addComputePerDof("v", "v+0.5*dt*f/m");
    ```

    The first step updates the velocities based on the current forces. The second step updates the positions based on the new velocities, and the third step updates the velocities again. Although the first and third steps look identical, the forces used in them are different. You do not need to tell the integrator that; it will recognize that the positions have changed and know to recompute the forces automatically.

    The above example has two problems. First, it does not respect distance constraints. To make the integrator work with constraints, you need to add extra steps to tell it when and how to apply them. Second, it never gives Forces an opportunity to update the context state. This should be done every time step so that, for example, an `AndersenThermostat` can randomize velocities or a `MonteCarloBarostat` can scale particle positions. You need to add a step to tell the integrator when to do this. The following example corrects both these problems, using the RATTLE algorithm to apply constraints:

    ```
    CustomIntegrator integrator(0.001);
    integrator.addPerDofVariable("x1", 0);
    integrator.addUpdateContextState();
    integrator.addComputePerDof("v", "v+0.5*dt*f/m");
    integrator.addComputePerDof("x", "x+dt*v");
    integrator.addComputePerDof("x1", "x");
    integrator.addConstrainPositions();
    integrator.addComputePerDof("v", "v+0.5*dt*f/m+(x-x1)/dt");
    integrator.addConstrainVelocities();
    ```

    `CustomIntegrator` can be used to implement multiple time step integrators. The following example shows an r-RESPA integrator. It assumes the quickly changing forces are in force group 0 and the slowly changing ones are in force group 1. It evaluates the “fast” forces four times as often as the “slow” forces.

    ```
    CustomIntegrator integrator(0.004);
    integrator.addComputePerDof("v", "v+0.5*dt*f1/m");
    for (int i = 0; i < 4; i++) {
        integrator.addComputePerDof("v", "v+0.5*(dt/4)*f0/m");
        integrator.addComputePerDof("x", "x+(dt/4)*v");
        integrator.addComputePerDof("v", "v+0.5*(dt/4)*f0/m");
    }
    integrator.addComputePerDof("v", "v+0.5*dt*f1/m");
    ```

    The sequence of computations in a `CustomIntegrator` can include flow control in the form of “if” and “while” blocks. The computations inside an “if” block are executed either zero or one times, depending on whether a condition is true. The computations inside a “while” block are executed repeatedly for as long as the condition remains true. Be very careful when writing “while” blocks; there is nothing to stop you from creating an infinite loop!

    For example, suppose you are writing a Monte Carlo algorithm. Assume you have already computed a new set of particle coordinates “xnew” and a step acceptance probability “acceptanceProbability”. The following lines use an “if” block to decide whether to accept the step, and if it is accepted, store the new positions into “x”.

    ```
    integrator.beginIfBlock("uniform < acceptanceProbability");
    integrator.computePerDof("x", "xnew");
    integrator.endBlock();
    ```

    The condition in an “if” or “while” block is evaluated globally, so it may only involve global variables, not per-DOF ones. It may use any of the following comparison operators: =, <. >, !=, <=, >=. Blocks may be nested inside each other.

    An `Integrator` has one other job in addition to evolving the equations of motion: it defines how to compute the kinetic energy of the system. Depending on the integration method used, simply summing mv

    Call `setKineticEnergyExpression()` to set an expression for the kinetic energy. It is computed for every degree of freedom (excluding ones whose mass is 0) and the result is summed. The default expression is “m\*v\*v/2”, which is correct for many integrators.

    As example, the following line defines the correct way to compute kinetic energy when using a leapfrog algorithm:

    ```
    integrator.setKineticEnergyExpression("m*v1*v1/2; v1=v+0.5*dt*f/m");
    ```

    The kinetic energy expression may depend on the following pre-defined variables: x, v, f, m, dt. It also may depend on user-defined global and per-DOF variables, and on the values of adjustable parameters defined in the integrator’s `Context`. It may

    Expressions may involve the operators + (add), - (subtract), \* (multiply), / (divide), and ^ (power), and the following functions: sqrt, exp, log, sin, cos, sec, csc, tan, cot, asin, acos, atan, sinh, cosh, tanh, erf, erfc, min, max, abs, floor, ceil, step, delta, select. All trigonometric functions are defined in radians, and log is the natural logarithm. step(x) = 0 if x is less than 0, 1 otherwise. delta(x) = 1 if x is 0, 0 otherwise. select(x,y,z) = z if x = 0, y otherwise. An expression may also involve intermediate quantities that are defined following the main expression, using ”;” as a separator.

    Methods

    |  |  |
    | --- | --- |
    | `CustomIntegrator` | Create a `CustomIntegrator`. |
    | `getNumGlobalVariables` | Get the number of global variables that have been defined. |
    | `getNumPerDofVariables` | Get the number of per-DOF variables that have been defined. |
    | `getNumComputations` | Get the number of computation steps that have been added. |
    | `addGlobalVariable` | Define a new global variable. |
    | `getGlobalVariableName` | Get the name of a global variable. |
    | `addPerDofVariable` | Define a new per-DOF variable. |
    | `getPerDofVariableName` | Get the name of a per-DOF variable. |
    | `getGlobalVariable` | Get the current value of a global variable. |
    | `getGlobalVariableByName` | Get the current value of a global variable, specified by name. |
    | `setGlobalVariable` | Set the value of a global variable. |
    | `setGlobalVariableByName` | Set the value of a global variable, specified by name. |
    | `getPerDofVariable` | Get the value of a per-DOF variable. |
    | `getPerDofVariableByName` | Get the value of a per-DOF variable, specified by name. |
    | `setPerDofVariable` | Set the value of a per-DOF variable. |
    | `setPerDofVariableByName` | Set the value of a per-DOF variable, specified by name. |
    | `addComputeGlobal` | Add a step to the integration algorithm that computes a global value. |
    | `addComputePerDof` | Add a step to the integration algorithm that computes a per-DOF value. |
    | `addComputeSum` | Add a step to the integration algorithm that computes a sum over degrees of freedom. |
    | `addConstrainPositions` | Add a step to the integration algorithm that updates particle positions so all constraints are satisfied. |
    | `addConstrainVelocities` | Add a step to the integration algorithm that updates particle velocities so the net velocity along all constraints is 0. |
    | `addUpdateContextState` | Add a step to the integration algorithm that allows Forces to update the context state. |
    | `beginIfBlock` | Add a step which begins a new “if” block. |
    | `beginWhileBlock` | Add a step which begins a new “while” block. |
    | `endBlock` | Add a step which marks the end of the most recently begun “if” or “while” block. |
    | `getComputationStep` | Get the details of a computation step that has been added to the integration algorithm. |
    | `getKineticEnergyExpression` | Get the expression to use for computing the kinetic energy. |
    | `setKineticEnergyExpression` | Set the expression to use for computing the kinetic energy. |
    | `getRandomNumberSeed` | Get the random number seed. |
    | `setRandomNumberSeed` | Set the random number seed. |
    | `step` | Advance a simulation through time by taking a series of time steps. |

    Enum: ComputationType

    |  |  |
    | --- | --- |
    | **ComputeGlobal** | Compute an expression and store it in a global variable. |
    | **ComputePerDof** | Compute an expression for every degree of freedom and store it in a per-DOF variable. |
    | **ComputeSum** | Compute an expression for every degree of freedom, sum the values, and store the result in a global variable. |
    | **ConstrainPositions** | Update particle positions so all constraints are satisfied. |
    | **ConstrainVelocities** | Update particle velocities so the net velocity along all constraints is 0. |
    | **UpdateContextState** | Allow Forces to update the context state. |
    | **IfBlockStart** | Begin an “if” block. |
    | **WhileBlockStart** | Begin a while” block. |
    | **BlockEnd** | End an “if” or “while” block. |

    `CustomIntegrator`(double *stepSize*)¶
    :   Create a `CustomIntegrator`.

        |  |  |
        | --- | --- |
        | Parameters: |  |

        - **stepSize** – the step size with which to integrate the system (in picoseconds)

    int `getNumGlobalVariables`() *const*¶
    :   Get the number of global variables that have been defined.

    int `getNumPerDofVariables`() *const*¶
    :   Get the number of per-DOF variables that have been defined.

    int `getNumComputations`() *const*¶
    :   Get the number of computation steps that have been added.

    int `addGlobalVariable`(*const* std::string &*name*, double *initialValue*)¶
    :   Define a new global variable.

        |  |  |
        | --- | --- |
        | Parameters: |  |

        - **name** – the name of the variable
        - **initialValue** – the variable will initially be set to this value

        |  |  |
        | --- | --- |
        | Returns: | the index of the variable that was added |

    *const* std::string &`getGlobalVariableName`(int *index*) *const*¶
    :   Get the name of a global variable.

        |  |  |
        | --- | --- |
        | Parameters: |  |

        - **index** – the index of the variable to get

        |  |  |
        | --- | --- |
        | Returns: | the name of the variable |

    int `addPerDofVariable`(*const* std::string &*name*, double *initialValue*)¶
    :   Define a new per-DOF variable.

        |  |  |
        | --- | --- |
        | Parameters: |  |

        - **name** – the name of the variable
        - **initialValue** – the variable will initially be set to this value for all degrees of freedom

        |  |  |
        | --- | --- |
        | Returns: | the index of the variable that was added |

    *const* std::string &`getPerDofVariableName`(int *index*) *const*¶
    :   Get the name of a per-DOF variable.

        |  |  |
        | --- | --- |
        | Parameters: |  |

        - **index** – the index of the variable to get

        |  |  |
        | --- | --- |
        | Returns: | the name of the variable |

    double `getGlobalVariable`(int *index*) *const*¶
    :   Get the current value of a global variable.

        |  |  |
        | --- | --- |
        | Parameters: |  |

        - **index** – the index of the variable to get

        |  |  |
        | --- | --- |
        | Returns: | the current value of the variable |

    double `getGlobalVariableByName`(*const* std::string &*name*) *const*¶
    :   Get the current value of a global variable, specified by name.

        |  |  |
        | --- | --- |
        | Parameters: |  |

        - **name** – the name of the variable to get

        |  |  |
        | --- | --- |
        | Returns: | the current value of the parameter |

    void `setGlobalVariable`(int *index*, double *value*)¶
    :   Set the value of a global variable.

        |  |  |
        | --- | --- |
        | Parameters: |  |

        - **index** – the index of the variable to set
        - **value** – the new value of the variable

    void `setGlobalVariableByName`(*const* std::string &*name*, double *value*)¶
    :   Set the value of a global variable, specified by name.

        |  |  |
        | --- | --- |
        | Parameters: |  |

        - **name** – the name of the variable to set
        - **value** – the new value of the variable

    void `getPerDofVariable`(int *index*, std::vector<Vec3> &*values*) *const*¶
    :   Get the value of a per-DOF variable.

        |  |  |
        | --- | --- |
        | Parameters: |  |

        - **index** – the index of the variable to get
        - **values** – the values of the variable for all degrees of freedom are stored into this

    void `getPerDofVariableByName`(*const* std::string &*name*, std::vector<Vec3> &*values*) *const*¶
    :   Get the value of a per-DOF variable, specified by name.

        |  |  |
        | --- | --- |
        | Parameters: |  |

        - **name** – the name of the variable to get
        - **values** – [out] the values of the variable for all degrees of freedom are stored into this

    void `setPerDofVariable`(int *index*, *const* std::vector<Vec3> &*values*)¶
    :   Set the value of a per-DOF variable.

        |  |  |
        | --- | --- |
        | Parameters: |  |

        - **index** – the index of the variable to set
        - **values** – the new values of the variable for all degrees of freedom

    void `setPerDofVariableByName`(*const* std::string &*name*, *const* std::vector<Vec3> &*values*)¶
    :   Set the value of a per-DOF variable, specified by name.

        |  |  |
        | --- | --- |
        | Parameters: |  |

        - **name** – the name of the variable to set
        - **values** – the new values of the variable for all degrees of freedom

    int `addComputeGlobal`(*const* std::string &*variable*, *const* std::string &*expression*)¶
    :   Add a step to the integration algorithm that computes a global value.

        |  |  |
        | --- | --- |
        | Parameters: |  |

        - **variable** – the global variable to store the computed value into
        - **expression** – a mathematical expression involving only global variables. In each integration step, its value is computed and stored into the specified variable.

        |  |  |
        | --- | --- |
        | Returns: | the index of the step that was added |

    int `addComputePerDof`(*const* std::string &*variable*, *const* std::string &*expression*)¶
    :   Add a step to the integration algorithm that computes a per-DOF value.

        |  |  |
        | --- | --- |
        | Parameters: |  |

        - **variable** – the per-DOF variable to store the computed value into
        - **expression** – a mathematical expression involving both global and per-DOF variables. In each integration step, its value is computed for every degree of freedom and stored into the specified variable.

        |  |  |
        | --- | --- |
        | Returns: | the index of the step that was added |

    int `addComputeSum`(*const* std::string &*variable*, *const* std::string &*expression*)¶
    :   Add a step to the integration algorithm that computes a sum over degrees of freedom.

        |  |  |
        | --- | --- |
        | Parameters: |  |

        - **variable** – the global variable to store the computed value into
        - **expression** – a mathematical expression involving both global and per-DOF variables. In each integration step, its value is computed for every degree of freedom. Those values are then added together, and the sum is stored in the specified variable.

        |  |  |
        | --- | --- |
        | Returns: | the index of the step that was added |

    int `addConstrainPositions`()¶
    :   Add a step to the integration algorithm that updates particle positions so all constraints are satisfied.

        |  |  |
        | --- | --- |
        | Returns: | the index of the step that was added |

    int `addConstrainVelocities`()¶
    :   Add a step to the integration algorithm that updates particle velocities so the net velocity along all constraints is 0.

        |  |  |
        | --- | --- |
        | Returns: | the index of the step that was added |

    int `addUpdateContextState`()¶
    :   Add a step to the integration algorithm that allows Forces to update the context state.

        |  |  |
        | --- | --- |
        | Returns: | the index of the step that was added |

    int `beginIfBlock`(*const* std::string &*condition*)¶
    :   Add a step which begins a new “if” block.

        |  |  |
        | --- | --- |
        | Parameters: |  |

        - **condition** – a mathematical expression involving a comparison operator and global variables. All steps between this one and the end of the block are executed only if the condition is true.

        |  |  |
        | --- | --- |
        | Returns: | the index of the step that was added |

    int `beginWhileBlock`(*const* std::string &*condition*)¶
    :   Add a step which begins a new “while” block.

        |  |  |
        | --- | --- |
        | Parameters: |  |

        - **condition** – a mathematical expression involving a comparison operator and global variables. All steps between this one and the end of the block are executed repeatedly as long as the condition remains true.

        |  |  |
        | --- | --- |
        | Returns: | the index of the step that was added |

    int `endBlock`()¶
    :   Add a step which marks the end of the most recently begun “if” or “while” block.

        |  |  |
        | --- | --- |
        | Returns: | the index of the step that was added |

    void `getComputationStep`(int *index*, ComputationType &*type*, std::string &*variable*, std::string &*expression*) *const*¶
    :   Get the details of a computation step that has been added to the integration algorithm.

        |  |  |
        | --- | --- |
        | Parameters: |  |

        - **index** – the index of the computation step to get
        - **type** – [out] the type of computation this step performs
        - **variable** – [out] the variable into which this step stores its result. If this step does not store a result in a variable, this will be an empty string.
        - **expression** – [out] the expression this step evaluates. If this step does not evaluate an expression, this will be an empty string.

    *const* std::string &`getKineticEnergyExpression`() *const*¶
    :   Get the expression to use for computing the kinetic energy. The expression is evaluated for every degree of freedom. Those values are then added together, and the sum is reported as the current kinetic energy.

    void `setKineticEnergyExpression`(*const* std::string &*expression*)¶
    :   Set the expression to use for computing the kinetic energy. The expression is evaluated for every degree of freedom. Those values are then added together, and the sum is reported as the current kinetic energy.

    int `getRandomNumberSeed`() *const*¶
    :   Get the random number seed. See `setRandomNumberSeed()` for details.

    void `setRandomNumberSeed`(int *seed*)¶
    :   Set the random number seed. The precise meaning of this parameter is undefined, and is left up to each `Platform` to interpret in an appropriate way. It is guaranteed that if two simulations are run with different random number seeds, the sequence of random numbers will be different. On the other hand, no guarantees are made about the behavior of simulations that use the same seed. In particular, Platforms are permitted to use non-deterministic algorithms which produce different results on successive runs, even if those runs were initialized identically.

        If seed is set to 0 (which is the default value assigned), a unique seed is chosen when a `Context` is created from this `Force`. This is done to ensure that each `Context` receives unique random seeds without you needing to set them explicitly.

    void `step`(int *steps*)¶
    :   Advance a simulation through time by taking a series of time steps.

        |  |  |
        | --- | --- |
        | Parameters: |  |

        - **steps** – the number of time steps to take

High performance molecular simulation on GPUs


### Navigation

- Core Objects
- Forces
- Integrators
- Extras

©2015, Stanford University and the Authors.
|
Powered by Sphinx 1.3.1
& Alabaster 0.7.7
|
Page source
